# A Herpesviral Immediate Early Protein Promotes Transcription Elongation of Viral Transcripts

**DOI:** 10.1128/mBio.00745-17

**Published:** 2017-06-13

**Authors:** Hannah L. Fox, Jill A. Dembowski, Neal A. DeLuca

**Affiliations:** Department of Microbiology and Molecular Genetics, University of Pittsburgh School of Medicine, Pittsburgh, Pennsylvania, USA; University of Michigan—Ann Arbor

**Keywords:** RNA polymerases, herpes simplex virus, immediate early proteins, transcriptional regulation

## Abstract

Herpes simplex virus 1 (HSV-1) genes are transcribed by cellular RNA polymerase II (RNA Pol II). While four viral immediate early proteins (ICP4, ICP0, ICP27, and ICP22) function in some capacity in viral transcription, the mechanism by which ICP22 functions remains unclear. We observed that the FACT complex (comprised of SSRP1 and Spt16) was relocalized in infected cells as a function of ICP22. ICP22 was also required for the association of FACT and the transcription elongation factors SPT5 and SPT6 with viral genomes. We further demonstrated that the FACT complex interacts with ICP22 throughout infection. We therefore hypothesized that ICP22 recruits cellular transcription elongation factors to viral genomes for efficient transcription elongation of viral genes. We reevaluated the phenotype of an ICP22 mutant virus by determining the abundance of all viral mRNAs throughout infection by transcriptome sequencing (RNA-seq). The accumulation of almost all viral mRNAs late in infection was reduced compared to the wild type, regardless of kinetic class. Using chromatin immunoprecipitation sequencing (ChIP-seq), we mapped the location of RNA Pol II on viral genes and found that RNA Pol II levels on the bodies of viral genes were reduced in the ICP22 mutant compared to wild-type virus. In contrast, the association of RNA Pol II with transcription start sites in the mutant was not reduced. Taken together, our results indicate that ICP22 plays a role in recruiting elongation factors like the FACT complex to the HSV-1 genome to allow for efficient viral transcription elongation late in viral infection and ultimately infectious virion production.

## IMPORTANCE

HSV-1 interacts with many cellular proteins throughout productive infection. Here, we demonstrate the interaction of a viral protein, ICP22, with a subset of cellular proteins known to be involved in transcription elongation. We determined that ICP22 is required to recruit the FACT complex and other transcription elongation factors to viral genomes and that in the absence of ICP22 viral transcription is globally reduced late in productive infection, due to an elongation defect. This insight defines a fundamental role of ICP22 in HSV-1 infection and elucidates the involvement of cellular factors in HSV-1 transcription.

## INTRODUCTION

During productive infection, herpes simplex virus 1 (HSV-1) transcription is complexly regulated by both viral and cellular factors. Genes are transcribed by cellular RNA polymerase II (RNA Pol II) (1) in an ordered cascade with four classes: immediate early (α), early (β), leaky late (γ1), and true late (γ2) genes (2, 3). Four of the five immediate early proteins, ICP4, ICP0, ICP27, and ICP22, are involved in the regulation of viral transcription. ICP4 is a transcription factor that recruits cellular complexes, including TFIID and mediator, to viral genes to enhance transcription initiation and can also function to repress transcription of some viral genes (4–6). ICP0 is an E3 ubiquitin ligase (7) and plays a role in counteracting the repression of incoming viral genomes by host factors (8, 9) and abrogating the interferon response to infection (10, 11). ICP27 is involved in the nuclear export of viral mRNAs and has a role in recruiting RNA Pol II to viral genes (12–14). The immediate early protein ICP22 has been implicated in the promotion of late gene transcription, but the mechanism by which it functions is unknown (15–18).

ICP22 is a 420-amino-acid protein that is extensively posttranslationally modified, including phosphorylation by the viral protein UL13 (19–21). ICP22 is required for efficient productive infection *in vivo* and in fibroblast cells such as MRC5 cells. ICP22 mutants are notably less restricted in Vero, HeLa, and HEp-2 cell lines (15, 17, 22, 23). Studies of ICP22 mutants in restrictive cells have shown that in the absence of ICP22 the expression of certain late genes is reduced (15, 17, 18, 24, 25). Additionally, virions produced by an ICP22 mutant virus are abnormally assembled, possibly due to reduced levels of the products of the late genes that encode virion assembly and structural proteins (26).

ICP22 expression is associated with two effects on the cellular environment that may be related to its role in productive viral infection: the modification of RNA Pol II and the formation of VICE (virus-induced chaperone-enriched) domains (27). In uninfected cells, the C-terminal domain (CTD) of RNA Pol II is sequentially phosphorylated during cellular gene transcription. These modifications follow a pattern in which hypophosphorylated RNA Pol II is recruited to promoters and is then phosphorylated on serine-5 (Ser-5) of the CTD around the start of transcription initiation. As RNA Pol II proceeds along the gene, serine-2 (Ser-2) of the CTD is phosphorylated and Ser-5 phosphorylation gradually decreases toward the 3′ end of the gene. Phosphorylation of Ser-2 of the CTD is typically associated with the ability of RNA Pol II to overcome promoter-proximal pausing and facilitate elongation (28, 29). During infection with HSV-1, the CTD of RNA Pol II is uniquely modified: RNA Pol II exhibiting both Ser-5 and Ser-2 phosphorylation of the CTD is rapidly decreased in the cell, and RNA Pol II exhibiting only Ser-5 phosphorylation of the CTD accumulates. The alteration of CTD modifications requires ICP22 and has been hypothesized to affect viral transcription in some way (25, 30–32). VICE domains have been shown to sequester host protein chaperones (33) and to accumulate nascent proteins during HSV-1 infection (34).

Our laboratory recently developed a modified iPOND (isolation of proteins on nascent DNA) protocol to purify viral genomes and analyze proteins associated with these genomes during productive infection (35, 36). Many viral and cellular proteins were found to be associated with viral genomes, including ICP22 and a number of cellular proteins involved in transcription. Some transcription factors identified by this method have been previously shown to interact with ICP4, such as TFIID and mediator (37). The FACT transcription elongation complex was one of the most abundant protein complexes identified and was found to relocalize to viral replication compartments. The mechanism underlying this recruitment has not been determined (35, 36).

Preliminary experiments with viruses deficient for one or more immediate early proteins suggested that ICP22 may be involved in the recruitment of the FACT complex to viral genomes. To elucidate the role of ICP22 in HSV-1 productive infection, we undertook to further characterize the phenotype of an ICP22 mutant. In this study, we purified wild-type (wt) and ICP22 mutant viral genomes from infected cells to determine which proteins require ICP22 in order to associate with viral DNA. We found that the amounts of FACT complex subunits SSRP1 and Spt16 were substantially reduced on ICP22 mutant genomes. The FACT complex was originally identified for its role in transcription elongation through nucleosomes (38). We therefore sought to characterize the role of ICP22 in the recruitment of the FACT complex to viral genomes during productive HSV-1 infection and to investigate the transcriptional defects that occur in the absence of ICP22. We demonstrate that ICP22 physically interacts with the FACT complex and is essential for FACT complex and transcription elongation factor recruitment to viral DNA. Furthermore, in the absence of ICP22, RNA Pol II association with gene bodies was reduced while the association with transcription start site regions of viral genes was not affected. Taken together, our results indicate that ICP22 plays a role in recruiting elongation factors, including the FACT complex, to viral genomes to allow for efficient transcription elongation of viral genes.

## RESULTS

**FIG 1:**
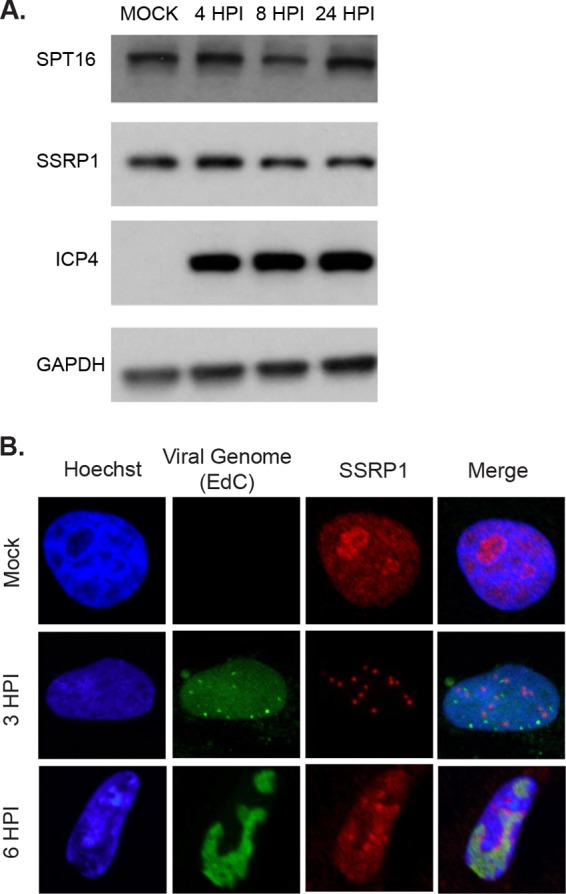
Abundance of FACT complex proteins and localization of SSRP1 in infected cells. (A) MRC5 cells were infected with wild-type (KOS) virus at an MOI of 10. Cell lysates were collected at the indicated hours postinfection. Cell lysates were run on a 10% SDS-PAGE gel and probed with antibodies against Spt16, SSRP1, ICP4, and GAPDH. (B) Vero cells were infected with unlabeled KOS, or KOS prelabeled with 10 μM EdC, at an MOI of 10. Cells infected with prelabeled KOS were fixed at 3 hpi. Cells infected with unlabeled KOS were incubated in medium containing 10 μM EdC starting at 4 hpi and then fixed at 6 hpi. Fixed cells were stained with Hoechst stain (blue) and reacted with Alexa Fluor azide as described in Materials and Methods to visualize viral DNA (green). SSRP1 was detected by immunofluorescence (red).

Previously, our lab has reported that the FACT complex is abundant on HSV-1 genomes during viral replication (35). Additionally, SSRP1 is redistributed within the nucleus upon infection in KOS-infected cells compared to mock-infected cells, although Spt16 and SSRP1 protein levels are not altered by infection (35) (Fig. 1). In order to determine if any of the viral immediate early proteins were responsible for this redistribution and recruitment of the FACT complex to viral genomes, we used immunofluorescence to assay SSRP1 redistribution in cells infected with a panel of viruses deficient for one or more immediate early proteins (see Table S1 in the supplemental material). We determined that SSRP1 was not redistributed upon infection with viruses deficient for ICP22. We therefore hypothesized that ICP22 may play a role in recruiting the FACT complex to viral genomes and that, by defining the relationship between ICP22 and the FACT complex, we could elucidate the role of ICP22 in productive infection.

10.1128/mBio.00745-17.3 TABLE S1  Analysis of SSRP1 localization in IE mutants. Vero cells were infected with viruses defective in one or more HSV-1 immediate early proteins. Infected cells were fixed and stained 3 h postinfection. Images were analyzed to determine whether SSRP1 reorganized into puncta (YES) or remained mostly in the nucleolus (NO). Virus references are included in Materials and Methods. Download TABLE S1, PDF file, 0.1 MB. Copyright © 2017 Fox et al. 2017 Fox et al. This content is distributed under the terms of the Creative Commons Attribution 4.0 International license.

### Redistribution of FACT complex subunit SSRP1 requires ICP22 expression.

**FIG 2:**
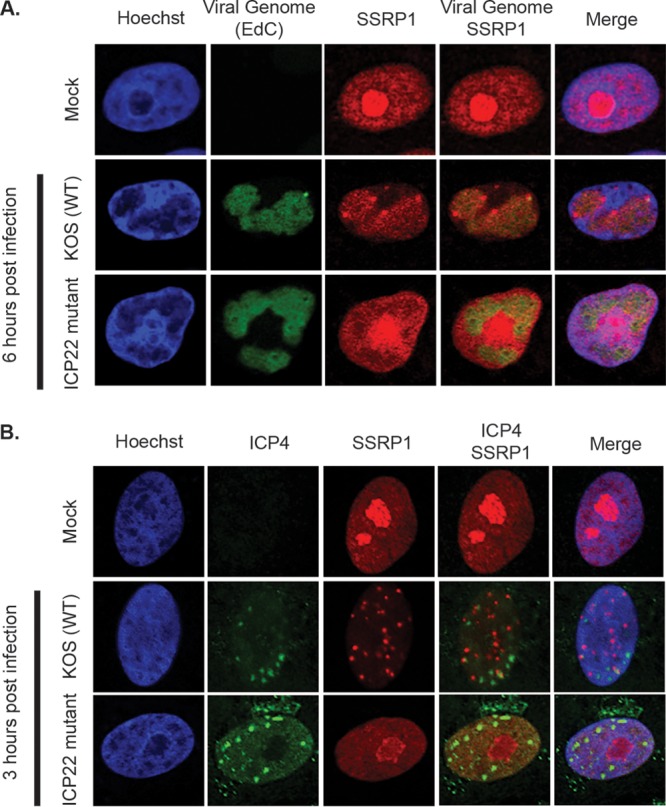
Effect of ICP22 on the localization of SSRP1 in HSV-1-infected cells. Vero cells were infected with wild-type (KOS) or ICP22 mutant (n199) virus at an MOI of 10. (A) EdC (10 μM) was added to the medium of infected cells from 4 to 6 hpi. The cells were then fixed, stained with Hoechst stain (blue), and reacted with Alexa Fluor azide as described in Materials and Methods to visualize viral DNA (green). SSRP1 was detected by immunofluorescence (red). Single cells shown are representative of cells in two independent replicates. (B) Infected cells were fixed at 3 hpi and stained with Hoechst stain (blue). ICP4 (green) and SSRP1 (red) were detected by immunofluorescence. Single cells shown are representative of cells in two independent replicates.

Immunofluorescence was used to further characterize the redistribution of the FACT complex relative to viral genomes as a function of ICP22. Vero cells were infected with either wild-type virus (KOS) or ICP22 mutant virus (n199), and cultures were incubated with the nucleoside analogue in the medium from 4 to 6 h postinfection (hpi). 5-Ethynyl-2′-deoxycytidine (EdC) labeling of replicating viral genomes enables tagging of viral DNA with Alexa Fluor via click chemistry (35, 36). The cultures were fixed at 6 hpi, labeled viral genomes were tagged with Alexa Fluor, and SSRP1 was probed via indirect immunofluorescence. In mock-infected cells, SSRP1 was concentrated in the nucleolus (Fig. 2A), where it is known to play a role in RNA polymerase I-mediated transcription of rRNA (39). In KOS-infected cells, SSRP1 colocalized with viral genomes in replication compartments in addition to gathering in foci around viral replication compartments. In n199-infected cells, SSRP1 did not relocalize and was excluded from viral replication compartments.

Because ICP22 is an immediate early protein, we also used immunofluorescence to visualize the localization of SSRP1 early in infection. Vero cells were infected with KOS or n199, fixed at 3 hpi, and probed with antibodies specific for ICP4 and SSRP1. ICP4 was used to represent the location of viral DNA because it has been shown to colocalize with viral genomes throughout infection (35). In cells infected with KOS, SSRP1 was redistributed into discrete foci in the nucleus by 3 hpi (Fig. 2B), and these foci were distinct from the majority of ICP4-containing foci. In cells infected with the ICP22 mutant n199, SSRP1 remained concentrated in the nucleolus, resembling mock-infected cells. We additionally observed that in cells infected with viral mutants that express ICP22 but do not express ICP4 or other combinations of immediate early proteins (Table S1), SSRP1 is redistributed similarly to SSRP1 in wild-type-infected cells. The redistribution of SSRP1 in infected cells is therefore a specific function of ICP22. These data suggest that ICP22 affects the redistribution of SSRP1 in the cell throughout infection.

### ICP22 is necessary for the association of elongation factors with viral genomes.

In order to determine how ICP22 affects the association of the FACT complex and other viral and cellular factors with the viral genome, we purified replicating KOS and n199 viral DNA, along with associated proteins, from infected cells as previously described (35, 36). We infected MRC5 cells at a multiplicity of infection (MOI) of 10 PFU/cell with either n199 or KOS and incubated cells in the presence of EdC from 4 to 6 hpi to label replicating viral DNA. Infected cultures without EdC added to the medium served as a negative control. At 6 hpi, nuclei were harvested and EdC-labeled viral DNA was covalently tagged with biotin via click chemistry. Genomes were then purified on streptavidin beads, and associated proteins were identified by mass spectrometry. Many viral and cellular proteins were enriched on both the KOS and n199 viral genomes relative to unlabeled negative controls (Table S2).

10.1128/mBio.00745-17.4 TABLE S2  Raw mass spectrometry data for iPOND experiments. Download TABLE S2, XLSX file, 0.2 MB. Copyright © 2017 Fox et al. 2017 Fox et al. This content is distributed under the terms of the Creative Commons Attribution 4.0 International license.

The association of most viral and cellular proteins with viral genomes was comparable between KOS and n199 as indicated by the similar recoveries of spectral counts (SpC) (Table 1). Levels of viral DNA binding proteins ICP4, UL42, and ICP8 were very similar between KOS and n199 EdC-labeled samples. Furthermore, the viral replication proteins UL9 (origin binding protein) and UL30 (DNA polymerase), as well as cellular DNA repair proteins, were present at similar levels on KOS and n199 genomes. Transcription initiation factors were found to be present at similar levels on KOS and n199 genomes or were slightly increased on n199 genomes. Interestingly, the levels of a subset of cellular transcription factors were selectively reduced on n199 genomes relative to KOS genomes. Most striking was the relative deficiency of a number of cellular transcription elongation factors. Specifically, there was a reduction in Spt5, Spt6, and FACT complex members Spt16 and SSRP1, as well as a modest reduction in the levels of RNA Pol II subunits. Both Spt16 and SSRP1 levels were reduced more than 20-fold, providing evidence that ICP22 plays a role in recruiting the FACT complex to replicating viral genomes.

**TABLE 1:** Comparison of proteins copurifying with ICP22 mutant and wild-type viral genomes^a^

Protein	Spectral count by replicate and virus			
	Replicate 1		Replicate 2	
	KOS	n199	KOS	n199
Viral proteins				
ICP4	689	696	834	916
ICP22	60	2	63	3
ICP8	592	539	972	910
UL9	199	218	255	341
UL30	213	229	456	507
UL42	369	255	411	320
Cellular repair proteins				
XRCC5 (KU80)	89	88	101	130
XRCC6 (KU70)	62	66	55	65
RNA polymerase II subunits				
PolrA	35	17	102	82
PolrB	19	6	47	42
Transcription elongation factors				
Spt16	57	4	78	7
SSRP1	43	0	29	0
Spt5	0	0	32	10
Spt6	12	0	41	2
Cdk9	5	9	6	11
Transcription initiation factors				
TBP (TFIID)	0	0	6	5
TAF1 (TFIID)	0	0	5	12
TAF6 (TFIID)	0	10	2	8
ERCC2 (TFIIH)	6	13	8	13
ERCC3 (TFIIH)	3	13	12	32
MED12 (mediator)	13	20	43	62
MED14 (mediator)	8	26	41	92
MED23 (mediator)	23	50	60	112
MED 24 (mediator)	15	23	25	52

a MRC5 cells were infected with wild-type (KOS) or an ICP22 mutant (n199) at an MOI of 10 and were incubated with EdC-containing medium or negative-control medium (no EdC) from 4 to 6 hpi. At 6 hpi, nuclei were harvested and the DNA was purified as described in Materials and Methods. Proteins eluted from genomes were analyzed by mass spectrometry. Values indicate spectral counts determined by mass spectrometry.

### ICP22 interacts with FACT complex members SSRP1 and Spt16.

Results thus far demonstrated that ICP22 is required for the relocalization of the FACT complex in infected cells and its association with viral genomes. In order to determine if these observations involve an interaction between ICP22 and FACT complex components, we constructed a virus expressing N-terminally FLAG-tagged ICP22 in a KOS background (ICP22-FLAG) to use in immunofluorescence and coimmunoprecipitation experiments.

**FIG 3:**
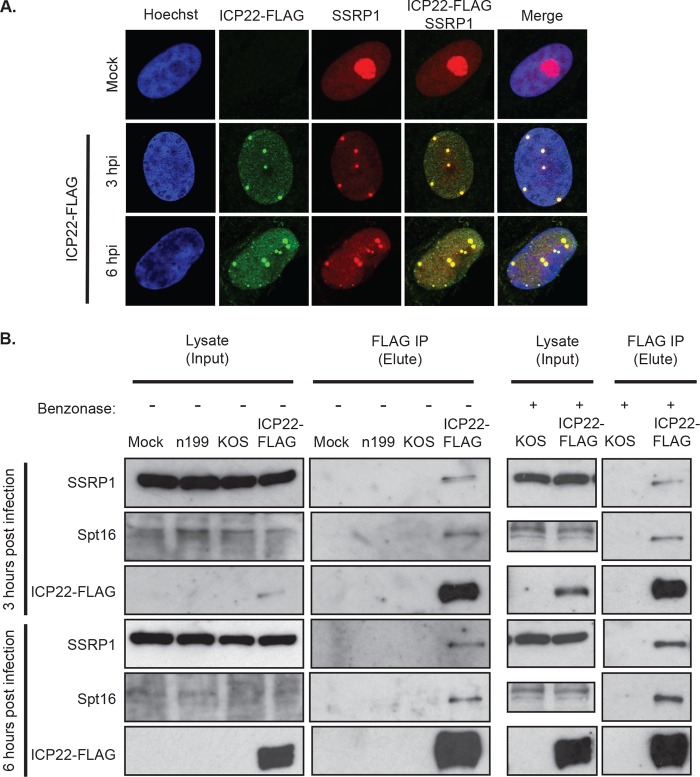
Interaction of ICP22 and the FACT complex in infected cells. (A) Localization of SSRP1 and ICP22-FLAG in infected cells. Vero cells were infected with virus expressing FLAG-tagged ICP22 protein (ICP22-FLAG) at an MOI of 10. Cells were fixed at 3 and 6 hpi and stained with Hoechst stain (blue). ICP22-FLAG (green) and SSRP1 (red) were detected by immunofluorescence. Single cells shown are representative of cells in two independent replicates. (B) Isolation of FLAG-tagged ICP22 and associated proteins in infected cells. MRC5 cells were infected with ICP22-FLAG at an MOI of 10. Cells were harvested at 3 or 6 hpi. Lysates were treated with (+) or without (-) Benzonase and bound to anti-FLAG beads. Proteins bound to the beads were eluted with 3×FLAG peptide and run on an SDS-PAGE gel. Gels were immunoblotted with anti-SSRP1, anti-Spt16, and anti-FLAG.

As in wt virus-infected cells, SSRP1 was relocalized from the pattern seen in mock-infected cells to discrete nuclear foci in ICP22-FLAG-infected cells (Fig. 3A). Furthermore, FLAG-tagged ICP22 colocalized with SSRP1. The number of SSRP1 foci in ICP22-FLAG-infected cells in Fig. 3A is fewer than the number of foci seen in Fig. 2B in wt virus-infected cells. While this may indicate that the relocalization is delayed in ICP22-FLAG-infected cells, we believe that this is most likely due to cell-to-cell variation.

We therefore immunoprecipitated ICP22-FLAG to determine if FACT complex members physically interact with ICP22. We infected cells with ICP22-FLAG, lysed cells at 3 or 6 hpi, and immunoprecipitated ICP22-FLAG and associated proteins with anti-FLAG antibody-coated magnetic beads. Mock-, KOS-, and n199-infected cells were used as controls. Proteins were eluted from the beads with 3×FLAG peptide and subjected to Western blot analysis. Both of the FACT complex subunits, SSRP1 and Spt16, coprecipitated with ICP22. Treatment of purified complexes prior to immunoprecipitation with Benzonase, a nuclease that degrades all forms of DNA and RNA, did not disrupt the interactions between ICP22 and FACT complex members, indicating that the presence of nucleic acid in the sample did not contribute to coprecipitation (Fig. 3B). Taken together, these data indicate that ICP22 interacts with complex members either directly or as part of a complex.

### Viral mRNA and genome abundance in ICP22 mutant-infected cells.

**FIG 4:**
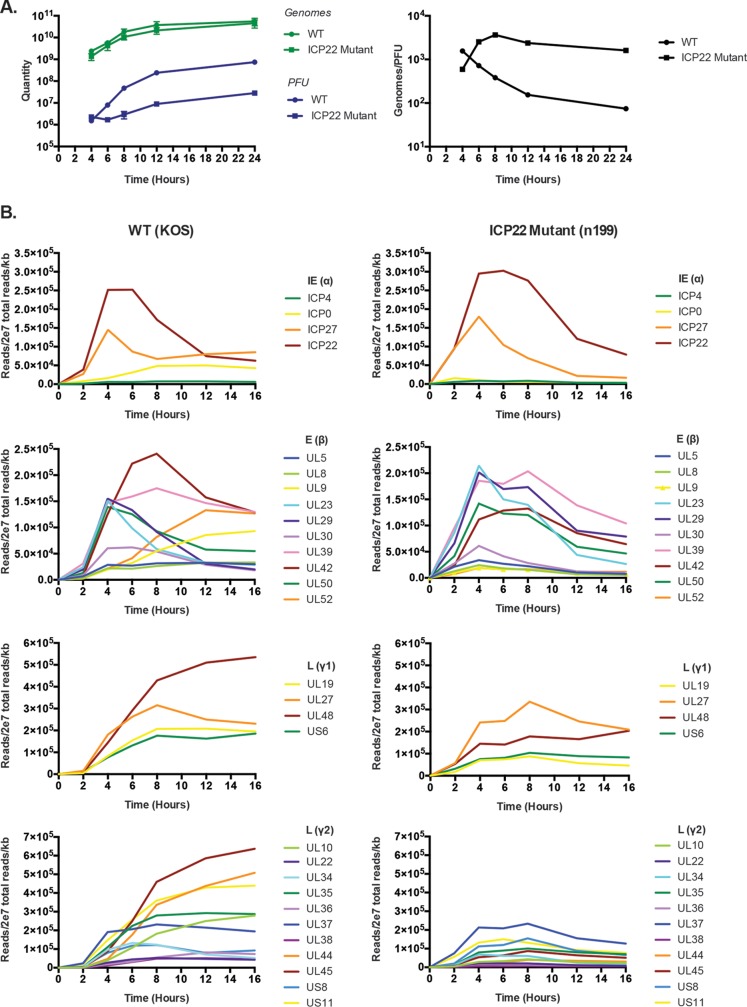
Replication and gene expression of an ICP22 mutant. (A) DNA replication and viral replication of ICP22 mutant n199 compared to wild-type KOS. MRC5 cells were infected and assayed at 4, 6, 8, 12, and 24 hpi. DNA replication was determined by quantitative PCR (genomes). Points are averages from 2 experiments; error bars represent standard deviations. Viral replication was determined by plaque assay (PFU). Points are averages from 2 experiments; error bars represent standard deviations. (B) RNA-seq analysis of select genes in all kinetic classes in ICP22 mutant (n199) compared to wild type (KOS). MRC5 cells were infected at an MOI of 5 and assayed at 2, 4, 6, 8, 12, and 16 hpi. Viral transcripts were quantified by RNA-seq as described in Materials and Methods.

To further investigate the significance of the interaction between the FACT complex and ICP22, we reevaluated the gene expression and DNA replication phenotypes of n199 (40) using more sensitive approaches. It has previously been reported that the rate of viral DNA replication of ICP22 mutant virus is similar to that of wild-type virus based on hybridization assays (17, 25, 40). To obtain a more quantitative account of viral DNA replication in the absence of ICP22, we used quantitative PCR to measure the number of viral genomes over time. We carried out infections in MRC5 cells, which are known to be restrictive to ICP22 mutant virus. One million cells were infected at an MOI of 5, and DNA was harvested throughout the productive viral replication cycle. DNA replication rates did not differ significantly between KOS and n199, indicating that ICP22 likely does not play a significant role in viral DNA replication (Fig. 4A). We also assessed infectious virus production throughout infection using plaque assays. We observed an almost 20-fold reduction in infectious virus produced from n199-infected cells compared to KOS-infected cells (Fig. 4A). These data suggest that although the n199 genome efficiently replicates, it has some defect or defects that prevent infectious virion production.

Previously, a reduction in late gene expression has been presumed to contribute to the reduced infectivity associated with n199. We therefore quantified viral mRNA abundance throughout infection to identify transcriptional defects with n199. Transcriptome sequencing (RNA-seq) was used to measure viral transcript levels at different times after infection with wt (KOS) or ICP22 mutant (n199) virus. RNA-seq data indicated that although late gene mRNA accumulation was indeed impaired in n199 as reported, the expression of viral genes in other classes was also affected (Fig. 4B). Reduced gene expression appears to depend more on the time postinfection than on the kinetic class of a particular gene. Specifically, the expression of viral genes that are increasingly expressed after 6 hpi in KOS was reduced in n199 compared to KOS, regardless of kinetic class. These genes include those previously identified to be less expressed in ICP22 mutant-infected cells, such as RL1, which codes for ICP0; US11 (18); UL48, which codes for VP16; UL44, which codes for gC; and UL36 (25). We also observed decreased expression of genes such as β genes UL9, UL42, UL50, and UL52; γ1 genes US6 and UL19; and γ2 genes UL45, UL35, and UL38, among others.

### Elongation of RNA polymerase transcription is impaired in an ICP22 mutant.

In ICP22 mutant-infected cells, there was a global reduction in the abundance of all viral transcripts late in infection (Fig. 4B). Furthermore, the main difference in the recruitment of cellular proteins to the n199 genome relative to KOS appears to be limited to factors involved in transcription elongation (Table 1). In order to determine if the reduced abundance of viral transcripts in the absence of ICP22 could be due to a defect in transcription elongation, we mapped the distribution of RNA Pol II on KOS and n199 genomes by chromatin immunoprecipitation sequencing (ChIP-seq) at 6 hpi, a critical time for the observed phenotypes. MRC5 cells were infected with either KOS or n199 and cross-linked at 6 hpi to capture the state of the genome at that time, and parallel immunoprecipitations were performed with antibodies specific for RNA Pol II and ICP4.

In wt HSV-1-infected cells, a unique form of RNA Pol II (IIi), in which the CTD is phosphorylated on Ser-5, accumulates. Additionally, reduction in the unphosphorylated form (IIa) and even greater reduction in the hyperphosphorylated form (IIo), in which the CTD is phosphorylated on both Ser-5 and Ser-2, are observed (30, 31). In ICP22 mutant virus-infected cells, the IIi form does not accumulate and there is greater abundance of the IIa form than in KOS-infected cells (25). We performed parallel immunoprecipitations with the two RNA Pol II antibodies 8WG16 and 4H8. 8WG16 recognizes both the IIa and the HSV-specific IIi forms of RNA Pol II, both of which are present in HSV-1-infected cells (30). 4H8 recognizes Ser-5-modified RNA Pol II and should therefore recognize IIi. Additionally, 4H8 recognizes RNA Pol II with both Ser-5 and Ser-2 phosphorylated because Ser-2 phosphorylation does not inhibit binding of the antibody to its Ser-5 epitope and should therefore recognize IIo (29). Although 8WG16 is more specific for IIa than 4H8, 4H8 can also recognize IIa. Both 8WG16 and 4H8 have been used in previous ChIP-seq studies to determine RNA Pol II levels on individual genes and have been found to detect similar patterns (41). Therefore, by using both 8WG16 and 4H8, we are able to detect all forms of RNA Pol II that have been identified to be present in KOS- and n199-infected cells (25).

Immunoprecipitated DNA was sequenced to determine the relative abundance of RNA Pol II and ICP4 across the viral genome. Consistent with results from the DNA replication assay (Fig. 4A), the total numbers of reads that map to the viral genome in the input samples were similar between KOS and n199 (Fig. S1A). Furthermore, consistent with mass spectrometry analysis (Table 1), the numbers of reads that map to the viral genome in ICP4 ChIP samples were similar between KOS and n199 and the numbers of reads that map to the viral genome in the RNA Pol II ChIP samples were somewhat reduced for n199 relative to KOS (Fig. S1A).

10.1128/mBio.00745-17.1 FIG S1  Total ChIP-seq reads. (A) Percentage of reads from n199 samples over KOS samples. Data for each replicate and averages from the two replicates. (B) Mapped reads on the HSV-1 genome for ChIP of ICP4, RNA Pol II 4H8, and RNA Pol II 8WG16 in n199- or KOS-infected cells. The maximums on the *y* axis are listed at the top of each panel. The number below is the total mapped reads. Download FIG S1, TIF file, 12.1 MB. Copyright © 2017 Fox et al. 2017 Fox et al. This content is distributed under the terms of the Creative Commons Attribution 4.0 International license.

The reads mapping across the viral genome for ICP4 and RNA Pol II are shown in Fig. S1B. The distribution of ICP4 across the viral genome was unaffected by mutation of ICP22. The details of ICP4 binding to the viral genome will be published elsewhere. At this level of resolution, the difference between the RNA Pol II binding profiles of n199 and KOS is quite subtle. The peaks in the n199 samples appear to be better defined with a possible reduction in read numbers between the peaks compared to the KOS samples. 8WG16 and 4H8 antibodies produced read patterns similar to one another and reveal the same differences in binding patterns between KOS and n199.

**FIG 5:**
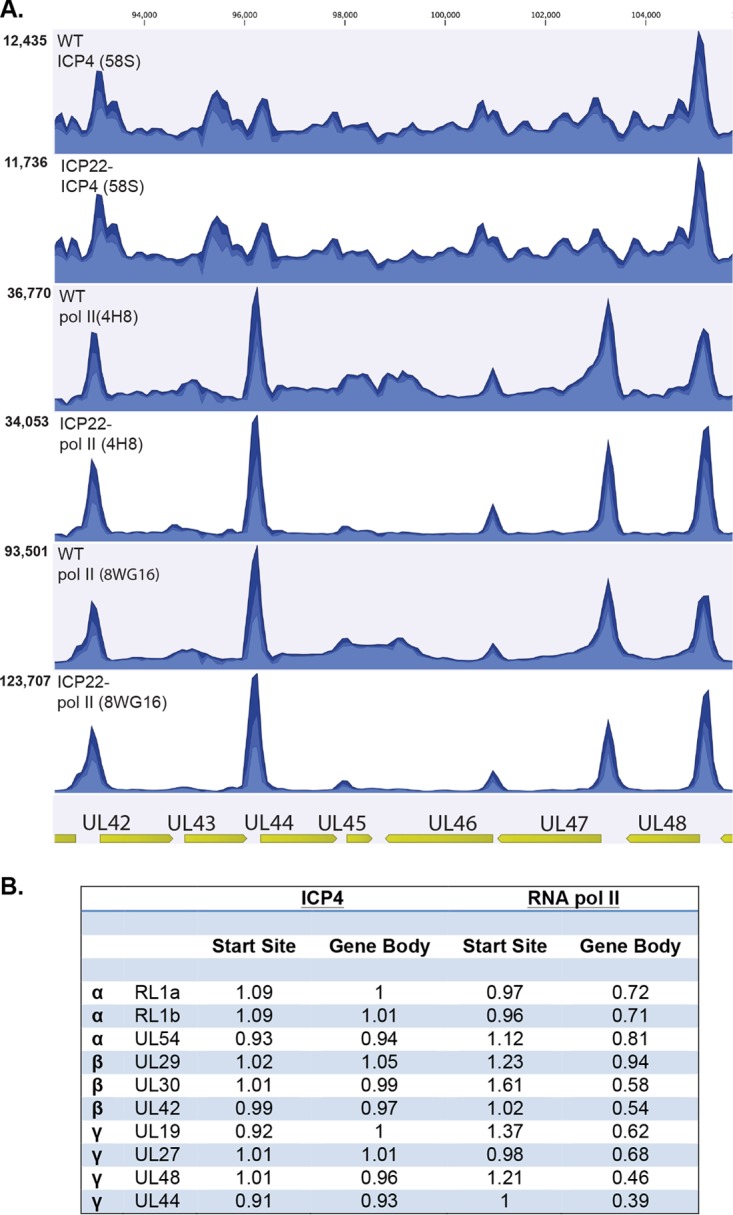
ChIP analysis of ICP4 and RNA Pol II on ICP22 mutant versus wt (KOS) genomes. MRC5 cells were infected with wild type (KOS) or ICP22 mutant (n199) at an MOI of 10. The infected cells were harvested and processed for ChIP-seq as described in Materials and Methods. (A) Mapped reads of the region between UL42 and UL48 on the genome for ChIP of ICP4, RNA Pol II (4H8), or RNA Pol II (8WG16) in n199- or KOS-infected cells. The maximums on the *y* axis are to the top left of each panel. Experiments were repeated twice, and the figure represents one data set. (B) Normalized ratio of n199 over KOS reads for start sites or gene bodies of representative IE, E, and L genes. The values used were averages from two experiments using the 4H8 antibody.

A higher-resolution representation of Fig. S1B in the region between UL42 and UL48 is shown in Fig. 5A. These genes are all expressed at 6 hpi in KOS-infected cells and represent members of the early through true late classes of genes. The numbers of reads in the RNA Pol II ChIP at or near transcription start sites were similar between KOS and n199. However, the numbers of reads mapping beyond the start site, in the message bodies, appeared to be significantly reduced in n199 (Fig. 5A).

In order to compare the relative abundance of Pol II and ICP4 on the start sites and message bodies of n199 and KOS, we calculated the ratio of normalized n199 to KOS reads for individual gene mRNA start sites and gene bodies of select genes representative of different kinetic classes for both ICP4 and RNA Pol II (Fig. 5B). For the calculations, we used the data from the 4H8 antibody as this antibody should detect the forms of RNA Pol II involved in the transcription of viral genes in KOS- and n199-infected cells, including Ser-5-phosphorylated RNA Pol II and RNA Pol II phosphorylated on both Ser-5 and Ser-2 (IIo). An example of the analysis is shown in Fig. S2. In the ICP4 immunoprecipitations, n199-over-KOS read ratios were consistently close to 1 for both gene start sites and gene bodies. On the other hand, the n199-over-KOS read ratios for the RNA Pol II immunoprecipitations were reduced on n199 viral gene bodies compared to KOS, particularly for genes whose mRNA levels were reduced later in infection in the ICP22 mutant (Fig. 4B). The number of RNA Pol II reads on transcription start sites was not reduced in n199 relative to KOS, with more reads on the n199 start sites in some cases (Fig. 5B). This observation supports a model in which transcription elongation is reduced in the absence of ICP22; however, transcription initiation is unaffected.

10.1128/mBio.00745-17.2 FIG S2  Calculation of start site and gene body reads for selected genes. A schematic of how the numbers in Fig. 5B were calculated using the gene UL44 as an example. Download FIG S2, TIF file, 12.2 MB. Copyright © 2017 Fox et al. 2017 Fox et al. This content is distributed under the terms of the Creative Commons Attribution 4.0 International license.

## DISCUSSION

HSV-1 genes are transcribed by cellular RNA Pol II (1). However, the Pol II machinery of the cell is perturbed or augmented by viral gene products, resulting in the selective and efficient expression of viral genes. For example, the immediate early protein ICP4 plays an important role in the regulation of transcription initiation. ICP4 binds to viral DNA and interacts with crucial cellular proteins involved in transcription initiation such as transcription factors, TFIID, and mediator (37, 42), facilitating their recruitment to the viral genome. In this study, we describe findings that shed light on how the immediate early protein ICP22 affects RNA Pol II transcription of viral genes, thus promoting the production of viral progeny. Specifically, ICP22 interacts with the FACT complex and is responsible for the relocation of FACT in the nucleus and its recruitment to the viral genome along with two other transcription elongation factors, Spt5 and Spt6. In addition, while ICP22 was not required for wt levels of RNA Pol II on the transcription start sites of viral genes, the amount of RNA Pol II in the bodies of viral genes was significantly reduced in the absence of ICP22. These observations suggest that ICP22 promotes the elongation of RNA Pol II transcription of viral genes late after infection, likely through the recruitment of transcription elongation factors.

The FACT complex is a histone chaperone that is known to dissociate H2A-H2B dimers from nucleosomes to allow access to DNA by elongating RNA Pol II and other factors (43, 44). The FACT complex is involved in cellular transcription elongation in addition to other processes in the cell such as DNA replication (45, 46). In addition to displacing nucleosomes by dissociating H2A-H2B dimers, the FACT complex can also destabilize nucleosomes globally, contributing to the maintenance of a loose chromatin structure (47). The FACT complex may therefore contribute to the establishment and maintenance of the loosely associated chromatin structure observed on HSV-1 genomes during viral DNA replication (48). Furthermore, the FACT complex has been found to be involved in the latent replication of Kaposi’s sarcoma-associated herpesvirus and in the efficient transcription of human cytomegalovirus immediate early genes (49, 50). In addition to the FACT complex, we discovered a substantial decrease in the amount of Spt6 and Spt5 recruited to viral genomes in the absence of ICP22 (Table 1). All of these proteins have established roles in cellular transcription elongation and are known to interact with RNA Pol II and one another (51–54). If these factors are not recruited to paused RNA Pol II, transcription arrest can occur (28).

Previously, it was determined that late gene transcription, as well as the transcription of the immediate early gene that codes for ICP0, is attenuated in ICP22 mutants (15, 17, 18, 26). However, the connection between the genes affected was not elucidated. It has been suggested that a common sequence may be responsible for uniting those genes affected by ICP22 (27). We used RNA-seq to characterize the transcription of viral genes in the ICP22 mutant and observed a global decrease in viral mRNA abundance beginning at 4 to 6 hpi, after the onset of viral DNA replication. Late genes are affected more drastically and noticeably because they are typically upregulated 4 to 6 hpi. Immediate early and early genes that are typically expressed at consistent or rising levels at 4 to 6 hpi, including ICP0, are impacted by the absence of ICP22 in a manner similar to late genes (Fig. 4B). Consistent with previous observations (17, 25, 40), we did not find a defect in viral DNA replication in the n199 background (Fig. 4A).

The observation that transcription elongation factors are reduced on ICP22 mutant genomes suggests that transcription elongation may be responsible for the inefficient viral gene expression in ICP22 mutants. This hypothesis is further supported by the ChIP-seq data demonstrating a specific reduction in the amount of RNA Pol II on the bodies of viral genes in the ICP22 mutant compared to wild-type virus, whereas that amount of RNA Pol II on their transcription start sites was relatively unaffected (Fig. 5).

The forms of RNA Pol II in HSV-infected cells are relevant to these observations. In uninfected cells, unphosphorylated RNA Pol II (IIa) is recruited to promoters, where it is phosphorylated on Ser-5 at the start of transcription initiation. Ser-2 phosphorylation of paused RNA Pol II results in the hyperphosphorylated form of RNA Pol II (IIo) (55). This form of RNA Pol II is closely associated with actively elongating cellular transcripts, and elongation factors such as Spt6 are known to bind to Ser-2-phosphorylated forms (56, 57). However, Ser-2-phosphorylated forms of Pol II are greatly depleted in wild-type and ICP22 mutant virus-infected cells (25, 30–32, 58). This raises the question of how transcription elongation occurs in infected cells with altered Pol II CTD phosphorylation.

The formation of the IIo form of RNA Pol II requires the action of cdk9. However, there is a lack of consensus on the role of phosphorylation of Ser-2 of the CTD by cdk9 in HSV-1 gene transcription. It has been postulated that ICP22 interacts with or recruits cdk9 in order to phosphorylate Ser-2 of the CTD (59, 60). There is evidence that ICP22 physically associates with cdk9 (59, 60) and that inhibition of kinases such as cdk9 can lead to decreased transcription during HSV-1 infection (61). Others have observed a rapid loss of RNA Pol II Ser-2 phosphorylation when ICP22 is expressed in cells either transiently or at later times during infection, indicating that Ser-2 phosphorylation by cdk9 may not be necessary for efficient viral transcription (24, 25, 31, 32). Moreover, it has been proposed that ICP22 interacts with cdk9 in order to inhibit it and prevent Ser-2 phosphorylation (62, 63). Supporting this is the observation that cellular transcripts are repressed during infection with HSV-1. Notably, this reduction in cellular transcription is enhanced late in infection (58). It may be that cdk9 and Ser-2 phosphorylation is more important early in infection and that late after infection ICP22 is required to sustain transcription elongation. In addition, persisting small amounts of Ser-2 phosphorylation in Vero cells may be a possible explanation for the reduced requirement of ICP22 in Vero cells relative to human fibroblasts. The possible temporal involvement of cdk9 in HSV infection and the basis for the differential requirement for ICP22 in different cell types remain to be tested.

We propose that ICP22 facilitates the recruitment of Spt5, Spt6, and the FACT complex to transcribing RNA Pol II independently of the form of Pol II involved in elongation in uninfected cells. Evidence exists that ICP22 interacts with RNA Pol II (61). The recruitment of the elongation factors through these interactions would then facilitate the elongation of viral transcripts, possibly by ensuring a looser chromatin structure on viral gene bodies. The precise nature of these interactions, the role of chromatin, and the implications for cellular transcription are under investigation.

## MATERIALS AND METHODS

### Cells and viruses.

Experiments were performed using MRC5 (human fetal lung) or Vero (African green monkey kidney) cells obtained from and propagated as recommended by ATCC. The viruses used in this study include wild-type KOS, 22/n199 (n199), and ICP22-FLAG. n199 is derived from wild-type KOS and contains a linker carrying stop codons in the US1 gene as has been previously described (40). ICP22-FLAG was generated using red-mediated recombination in a bacterial artificial chromosome (BAC) containing KOS DNA (64–66). A FLAG tag was inserted after the start codon of US1. Modified BAC constructs were then transfected into Vero cells using Lipofectamine 2000 transfection reagent (Life Technologies, Inc.). Virus produced was harvested, plaque purified, and sequenced to verify FLAG tag insertion. Additional viruses used include d109, d106, d92, and d99 (67); d95, d96, and DMP (68); d120 (6); and 5dl1.2 (69).

### Immunoblotting for protein abundance.

MRC5 cells were infected at an MOI of 10 by incubation with KOS virus in Tricine-buffered saline (TBS) for 1 h before inoculum was removed and cells were rinsed with TBS. Cells were then incubated at 37°C in Dulbecco’s modified Eagle’s medium (DMEM) containing 2% fetal bovine serum (FBS) for the indicated amount of time. Samples were rinsed with ice-cold TBS plus 0.1 mM tosyl-l-lysyl-chloromethane hydrochloride (TLCK) before being scraped into 2× SDS-PAGE sample buffer. Samples were boiled for 10 min before analysis. Samples were run on a 10% precast gel (Bio-Rad), transferred to a polyvinylidene fluoride membrane (Amersham), and probed with the following primary antibodies: rabbit anti-Spt16 H300 (Santa Cruz; 1:500), mouse anti-SSRP1 10D1 (BioLegend; 1:500), anti-ICP4 58S (1:500), and anti-glyceraldehyde-3-phosphate dehydrogenase (anti-GAPDH) Ambion AM4300. After incubation with anti-rabbit–horseradish peroxidase (HRP) or anti-mouse–HRP secondary antibody (Promega), bands were visualized using ECL Prime Western blot detection reagent (Amersham).

### Imaging of viral DNA and immunofluorescence.

Vero cells (2 × 10^5^) were plated on coverslips in 12-well dishes. Cells were infected at an MOI of 10 by incubation with virus in TBS for 1 h before inoculum was removed and cells were rinsed with TBS. Cells were then incubated at 37°C in Dulbecco’s modified Eagle’s medium (DMEM) containing 2% fetal bovine serum (FBS) for the indicated amount of time. Labeling of replicating genomes was carried out as described previously (35). Briefly, EdC (10 µM) was added to the medium of infected cells at 4 hpi and fixed at 6 hpi. EdC-labeled DNA was then visualized by conjugating EdC to Alexa Fluor 488 azide using the Click-iT Alexa Fluor 488 imaging kit (Thermo Fisher). The immunofluorescence assay was carried out as described previously (35) with the following primary antibodies: rabbit anti-ICP4 N15 (1:500), mouse anti-SSRP1 10D1 (BioLegend; 1:200), and mouse anti-FLAG M2 (Sigma; 1:5,000). Secondary antibodies used included goat anti-rabbit conjugated to Alexa Fluor 488 (Invitrogen; 1:500), goat anti-mouse conjugated to Alexa Fluor 594 (Invitrogen; 1:500), goat anti-mouse IgG2b conjugated to Alexa Fluor 594 (Invitrogen; 1:500), and goat anti-mouse IgG1 conjugated to Alexa Fluor 488 (Invitrogen; 1:500). Images were obtained using an Olympus FluoView FV1000 confocal microscope.

### Isolation of viral genomes and associated proteins.

Viral DNA isolation was carried out as described previously (36) with the following modifications. MRC5 cells were infected with KOS or n199 at an MOI of 10 and incubated at 37°C in DMEM containing 2% FBS for 4 h, at which time 10 μM EdC was added to the medium. Infections carried out in the absence of EdC were used as negative controls. At 6 hpi, nuclei were harvested and processed as described above. Genome-associated proteins were analyzed by mass spectrometry as described previously (35). Mass spectrometry was carried out by MSbioworks, LLC, Ann Arbor, MI.

### Coimmunoprecipitation and immunoblotting.

MRC5 cells were grown to confluence in a 150-mm plate. Cells were infected at an MOI of 10 by incubation with indicated virus or mock infection in TBS for 1 h before the inoculum was removed and cells were rinsed with TBS. Cells were then incubated at 37°C in DMEM containing 2% FBS for the indicated amount of time. After incubation, cells were rinsed with ice-cold TBS plus 0.1 mM tosyl-l-lysyl-chloromethane hydrochloride (TLCK). Cells were then lysed at 4°C for 30 min in 1.5 ml lysis buffer (50 mM Tris-HCl [pH 7.4], 300 mM NaCl, 1% Triton X-100, 1 mM EDTA, Roche protease inhibitor cocktail). Lysates were then centrifuged at 1.8 × 10^3^ × *g* for 10 min at 4°C, and supernatants were filtered through a 100-μm cell strainer (Corning). Indicated samples were incubated in 10 μl Benzonase (Novagen) at this step for 10 min. Samples were then combined 1:1 with binding buffer (50 mM Tris-HCl [pH 7.4], 1% Triton X-100, 1 mM EDTA, Roche protease inhibitor cocktail) and added to anti-FLAG M2 magnetic beads (Sigma). Immunoprecipitation was carried out according to the manufacturer’s protocol with a few modifications. Briefly, samples were bound to the anti-FLAG beads overnight and following a series of washes with Tris-buffered saline (50 mM Tris-HCl [pH 7.4], 150 mM NaCl, Roche protease inhibitor cocktail). Bound proteins were eluted using the recommended concentration of 3×FLAG peptide (Sigma). Samples were combined 1:1 with 2× SDS-PAGE sample buffer and boiled for 10 min before analysis. Samples were run on a 7.5% precast gel (Bio-Rad), transferred to a polyvinylidene fluoride membrane (Amersham), and probed with the following primary antibodies: rabbit anti-Spt16 H300 (Santa Cruz; 1:500), mouse anti-SSRP1 10D1 (BioLegend; 1:500), and mouse anti-FLAG M2 (Sigma; 1:500). After incubation with anti-rabbit–HRP or anti-mouse–HRP secondary antibody (Promega), bands were visualized using ECL Prime Western blot detection reagent (Amersham).

### Viral DNA quantification and growth curve.

MRC5 cells (1 × 10^6^) in 35-mm plates were infected with n199 or KOS at an MOI of 5. Cells were infected by incubation with indicated virus or mock infected in TBS for 1 h before removing inoculum and rinsing cells three times with warm TBS. Cells were then incubated at 37°C in DMEM containing 2% FBS for the indicated amount of time.

For DNA quantification, cells were lysed in 200 μl DNA lysis buffer (0.5% SDS, 100 mM NaCl, 400 μg/ml proteinase K), incubated at 37°C 4 h to overnight, incubated at 65°C for 30 min, and diluted 1:1,000 in 1× TE (100 mM Tris, 10 mM EDTA). Quantitative real-time PCR was carried out as described previously (70). Genome quantity was determined based on the amplification of the HSV-1 TK gene using the following primers: TkdsF1 (5′-ACC CGC TTA ACA GCG TCA ACA-3′) and TkdsR1 (5′-CCA AAG AGG TGC GGG AGT TT-3′). Standard curves were established using serially diluted purified KOS genomic DNA.

To assess infectious virus production, cells were harvested by scraping into incubation medium. Cells were then lysed by freeze-thaw and sonication, following which cells were pelleted and supernatant was isolated. Supernatant was serially diluted and used to infect Vero cell monolayers in 6-well plates. After infection, cells were incubated at 37°C in overlay medium (DMEM, 10 g/liter methylcellulose, 2% FBS). After 3 days, the medium was removed and cells were stained with 10 mg/ml crystal violet in 50% ethanol. PFU was determined as an average from two duplicate wells.

### RNA-seq.

MRC5 cells were seeded into 60-mm dishes at a density of 2 × 10^6^ cells per dish, infected with n199 or KOS at an MOI of 10 PFU/cell, and incubated at 37°C. At the appropriate time postinfection, RNA was isolated using the Ambion RNaqueous-4 PCR kit using the included protocol. The isolated total RNA was quantified using an Agilent eukaryotic RNA Nano kit and a 2100 Bioanalyzer. One microgram of total RNA from each sample was used to create sequencing libraries using the TruSeq RNA sample preparation kit (Illumina). Individual samples each with unit barcodes were quantified using an Agilent DNA 7500 chip and a 2100 Bioanalyzer. Pooled multiplexed samples were sequenced at the Tufts University genomics facility. The demultiplexed sequence read files were mapped to the HSV strain KOS genome and quantified using CLC Genomics Workbench.

### ChIP-seq.

MRC5 cells were seeded into 600-cm^2^ square tissue culture dishes at a density of 7 × 10^7^ cells per dish, infected with n199 or KOS at an MOI of 10 PFU/cell, overlaid with 100 ml medium, and incubated at 37°C. At 6 hpi, 5 ml of 20% formaldehyde was added to the culture medium. The cultures were incubated at room temperature for 5 min, followed by the addition of 5 ml of 2.5 M glycine. All subsequent procedures were performed at 4°C unless otherwise stated. The cultures were then washed with TBS and scraped into 50 ml of FLB {5 mM PIPES [piperazine-*N*,*N*′-bis(2-ethanesulfonic acid)], pH 8.0, 85 mM KCl, 0.5% Igepal CA-630 [US Biological], Roche protease inhibitor cocktail}. The cells were then pelleted by low-speed centrifugation, resuspended in 5 ml FLB, and pelleted again. The cell pellet was resuspended in 1.2 ml RIPA buffer (1× phosphate-buffered saline [PBS], 0.5% sodium deoxycholate, 0.1% sodium dodecyl sulfate, Roche protease inhibitor cocktail) and sonicated for 6 intervals of 30 s each on ice with a Sonics Vibra-Cell VCX 130 sonicator equipped with a 3-mm microprobe. The sonicated material was centrifuged at 1.8 × 10^3^ × *g* for 15 min. DNA was isolated from 50 µl of the supernatant as a total input control. The remainder was divided equally to use in immunoprecipitations using the ICP4 and RNA Pol II monoclonal antibodies, 58S and either 8WG16 (Abcam; ab817) or 4H8 (Abcam, Inc.; ab5408), respectively. Twenty-five microliters of monoclonal antibody was bound to 50 µl of Dynabeads M280 sheep anti-mouse IgG beads (Invitrogen) in 5% bovine serum albumin (BSA) in PBS overnight. The antibody-coated beads were extensively washed with 5% BSA in PBS and then added to the reserved sonicated extracts, which were incubated overnight. The immunoprecipitation mixtures were washed five times with LiCl wash buffer (100 mM Tris-HCl buffer, pH 7.5, 500 mM LiCl, 1.0% Igepal CA-630, 1.0% sodium deoxycholate) and once with TE. The beads were then resuspended in IP elution buffer (1.0% sodium dodecyl sulfate, 0.1 M NaHCO_3_) and incubated at 65°C for 2 h, after which time the beads were removed and incubation of the supernatant was continued overnight at 65°C to reverse cross-links. The samples were then extracted with phenol-chloroform-isoamyl alcohol (25:24:1) twice and with chloroform-isoamyl alcohol (24:1) once and then purified using Qiagen PCR cleanup columns. The quantity of DNA in each sample was quantified using a Qubit 2.0 fluorometer (Invitrogen). Ten nanograms of each sample was used to create sequencing libraries using the NEBNext Ultra II DNA library preparation kit (New England Biolabs). Individual samples each with unit barcodes were quantified using an Agilent DNA 7500 chip and a 2100 Bioanalyzer. Pooled multiplexed samples were sequenced at the Tufts University genomics facility. The demultiplexed sequence read files were mapped to the HSV strain KOS genome and analyzed using CLC Genomics Workbench.
