# Erratum for Fox et al., “A Herpesviral Immediate Early Protein Promotes Transcription Elongation of Viral Transcripts”

**DOI:** 10.1128/mBio.00255-18

**Published:** 2018-03-06

**Authors:** Hannah L. Fox, Jill A. Dembowski, Neal A. DeLuca

**Affiliations:** a Department of Microbiology and Molecular Genetics, University of Pittsburgh School of Medicine, Pittsburgh, Pennsylvania, USA

## ERRATUM

Volume 8, no. 3, e00745-17, 2017, https://doi.org/10.1128/mBio.00745-17. The following data availability information should have been included.

### Availability of data.

The data set for the RNA sequencing experiment is available in the GEO database under accession no. GSE109420. The data sets for the ChIP sequencing experiments are available in the SRA database under accession no. SRP131749.

