# Supplementary material for: A Herpesviral Immediate Early Protein Promotes Transcription Elongation of Viral Transcripts
Source: mBio. 2017 Jun 13;8(3):e00745-17. doi: 10.1128/mBio.00745-17 (PMC5472187; doi:10.1128/mBio.00745-17)
Supplement: TABLE S1 [file mbo003173347st1.pdf]

Virus	Genes Mutated (ICP)	SSRP1 Relocalization
d109	4, 27, **22**, 47, 0	NO
d106	4, 27, **22**, 47	NO
d95	4, 27, **22**	NO
d92	4, 27	YES
d96	4, **22**	NO
DMP	27, **22**	NO
d120	4	YES
5dl1.2	27	YES
d99	0	YES
n199	**22**	NO

Table S1. Analysis of SSRP1 localization in IE Mutants. Vero cells were infected with viruses defective in one or more HSV-1 immediate early protein. Infected cells were fixed and stained 3 hours post infection. Images were analyzed to determine whether SSRP1 reorganized into puncta (YES) or remained mostly in the nucleolus (NO).
Virus references are included in Materials and Methods.
