# Supplementary figures and images for: A Herpesviral Immediate Early Protein Promotes Transcription Elongation of Viral Transcripts
Source: mBio. 2017 Jun 13;8(3):e00745-17. doi: 10.1128/mBio.00745-17 (PMC5472187; doi:10.1128/mBio.00745-17)

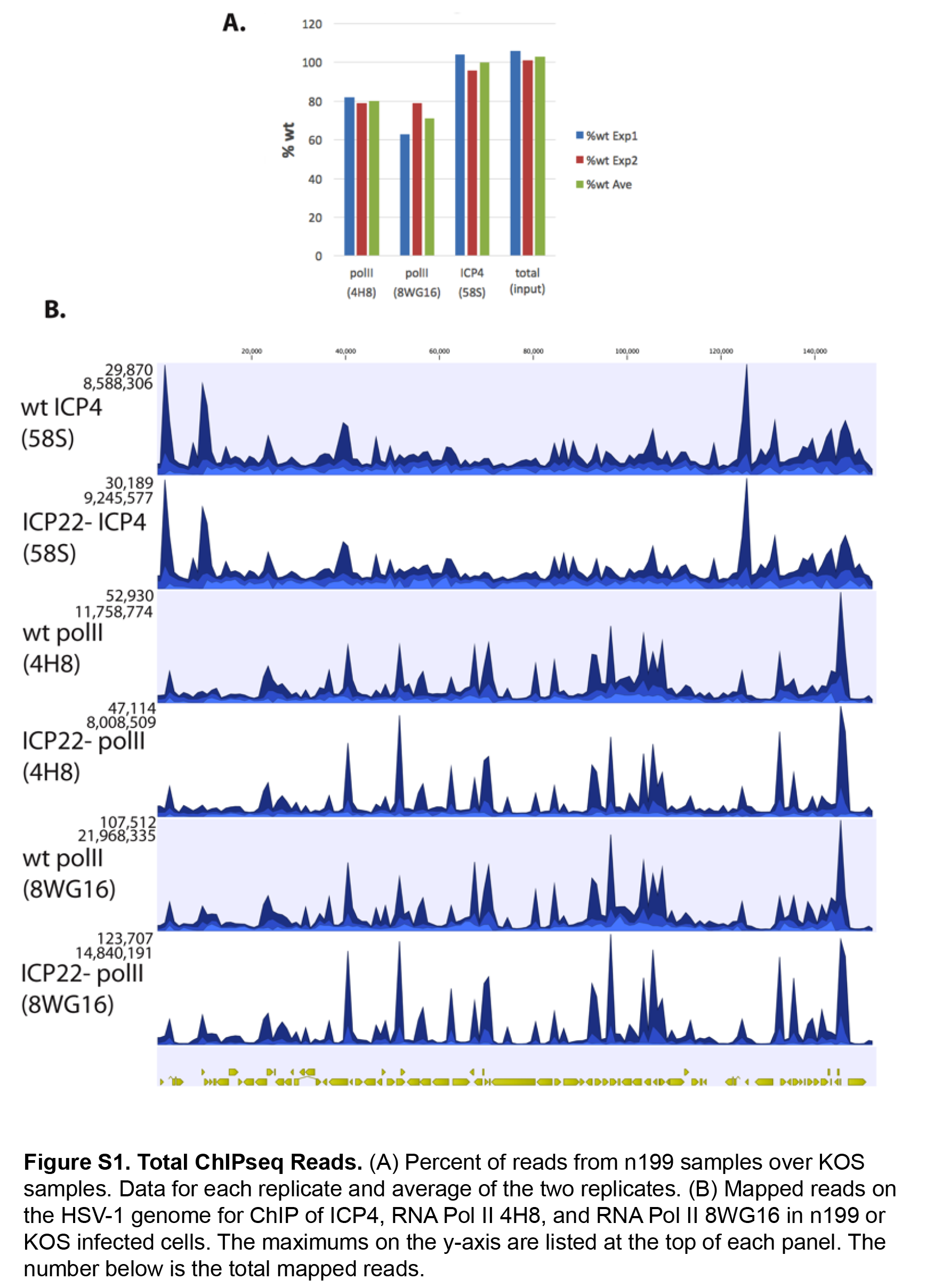

Supplement: FIG S1 [file mbo003173347sf1.tif]

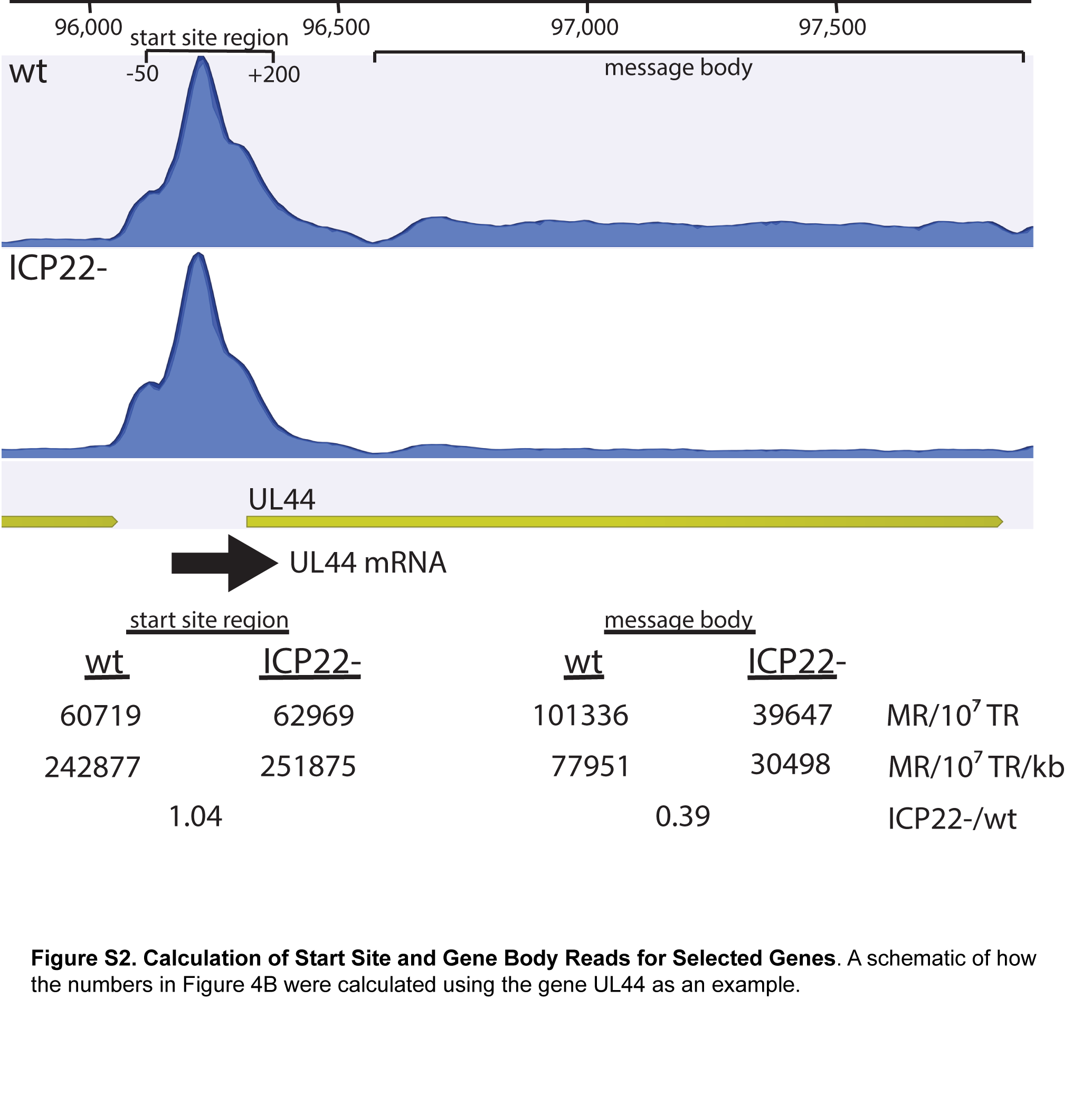

Supplement: FIG S2 [file mbo003173347sf2.tif]
